# Immunohistochemical analysis of tumor budding in stage II colon cancer: exploring zero budding as a prognostic marker

**DOI:** 10.1007/s00428-024-03860-2

**Published:** 2024-07-08

**Authors:** Maria Pihlmann Kristensen, Ulrik Korsgaard, Signe Timm, Torben Frøstrup Hansen, Inti Zlobec, Sanne Kjær-Frifeldt, Henrik Hager

**Affiliations:** 1 Department of Pathology, Lillebaelt Hospital, University Hospital of Southern Denmark, Vejle, Denmark; 2 Institute of Regional Health Research, University of Southern Denmark, Odense, Denmark; 3 Danish Colorectal Cancer Center South, Lillebaelt Hospital, University Hospital of Southern Denmark, Vejle, Denmark; 4 Department of Oncology, Lillebaelt Hospital, University Hospital of Southern Denmark, Vejle, Denmark; 5 Institute of Tissue Medicine and Pathology, University of Bern, Bern, Switzerland; 6 Department of Pathology, Aarhus University Hospital, Aarhus, Denmark

**Keywords:** Stage II colon cancer, Immunohistochemistry, Tumor budding, Survival, Scoring system

## Abstract

Tumor budding, a biomarker traditionally evaluated using hematoxylin and eosin (H&E) staining, has gained recognition as a prognostic biomarker for stage II colon cancer. Nevertheless, while H&E staining offers valuable insights, its limitations prompt the utilization of pan-cytokeratin immunohistochemistry (IHC). Consequently, this study seeks to evaluate the prognostic significance of tumor budding using IHC in a contemporary cohort of stage II colon cancer patients, aiming to deepen our understanding of this critical facet in cancer prognosis. We conducted a retrospective, population-based cohort study including 493 patients with stage II colon cancer and evaluated tumor budding using IHC, following the H&E-based guidelines proposed by the International Tumor Budding Consensus Conference Group. Correlation between H&E-based and IHC-based tumor budding was assessed using a four-tiered scoring system that included a zero budding (Bd0) category. Survival analyses explored the prognostic significance of tumor budding assessed by IHC and H&E. As expected, IHC-based tumor budding evaluation yielded significantly higher bud counts compared to H&E (*p* < 0.01). Interestingly, 21 patients were identified with no tumor budding using IHC. This was associated with significantly improved recurrence-free survival (HR = 5.19, *p* = 0.02) and overall survival (HR = 4.47, *p* = 0.04) in a multivariate analysis when compared to tumors with budding. The Bd0 category demonstrated a 100% predictive value for the absence of recurrence. In conclusion, IHC-based tumor budding evaluation in stage II colon cancer provides additional prognostic information. The absence of tumor budding is associated with a favorable prognosis and may serve as a potential marker for identifying patients with no risk of recurrence.

**Supplementary Information:**

The online version contains supplementary material available at 10.1007/s00428-024-03860-2.

## Introduction

Tumor budding has proven to be a prognostic biomarker in various cancers. High-grade tumor budding indicates disease progression and unfavorable survival outcomes [1] and is a consistent predictor of unfavorable prognosis and recurrence in stage II colon cancer [2–4]. The International Tumor Budding Consensus Conference (ITBCC) established a consensus on standardized definitions, scoring methods, and cut-off values for tumor budding in 2016 [5]. Since then, tumor budding has been incorporated as a histological prognostic factor in the 8th edition of the UICC TNM Classification [6], and the ITBCC recommendations have been validated in large cohorts of colorectal cancer [7, 8].

Although tumor budding is widely recognized among gastrointestinal pathologists, its significance as a prognostic marker is not universally accepted. The reluctance to report tumor budding scores stems from various factors, such as the extra time and effort needed for calculating the ITBCC score [9] and limited clinical demand, as high-grade tumor budding in isolation is not a biomarker warranting immediate recommendation of adjuvant chemotherapy. A tendency toward a beneficial effect of adjuvant chemotherapy has been demonstrated in intermediate and high-grade budding tumors; however, the results did not reach statistical significance [10]. Consequently, the lack of a convincing advantage of chemotherapy on survival in patients with a high grade of tumor budding remains a significant challenge in clinical practice. According to the American Society of Clinical Oncology (ASCO), high-grade tumor budding should be considered along with other high-risk factors in a shared decision-making process [11].

The implementation of tumor budding is also faced with interobserver variation, which directly influences the prognostic value of tumor budding [12], and various studies have highlighted the presence of variability among pathologists in determining the tumor budding score[12–14]. Pan-cytokeratin immunohistochemistry (IHC) has been proposed as a potential approach to mitigate interobserver variation [13, 15, 16] and enhance the precision of tumor budding assessment [17], thereby improving its clinical applicability.

The identification of tumor buds using routine hematoxylin and eosin (H&E) staining can be challenging due to inflammation and the presence of reactive inflammatory and stromal cells being misinterpreted as buds. The ITBCC recommends the use of a supporting cytokeratin in challenging cases to confirm that the counted cells are truly budding [5]. However, the final bud count should be performed on H&E [5], which is also in line with daily diagnostic practice from the participants in the Delphi consensus study [18]. Even so, additional cytokeratin staining has not demonstrated superiority over H&E alone [19], and therefore, more evidence is needed before considering IHC assessment of tumor budding in routine practice. The scoring criteria and cut-off values for high and low tumor budding would need to be defined independently from those based on H&E staining [16, 20, 21]. The use of pan-cytokeratin staining for the identification of budding cells was initially introduced by Prall et al. [20], evaluating tumor budding by examining a field of vision measuring 0.785 mm^2^, also suggested by the ITBCC guidelines. This densest high power field (HPF) approach has been confirmed to be effective in IHC-based tumor budding evaluation and comparable to the 10 HPF scoring method [22], which involves assessing the average number of buds and is commonly used for IHC-based prognostic analysis [23, 24].

Zlobec et al. proposed a “zero-budding” category for colon cancer that appears to be less aggressive than tumors with any degree of budding [25]. Some studies demonstrate that patients with zero budding have superior survival outcomes compared to those with even minimal budding [26, 27]. However, the zero-budding category has not previously been investigated or assessed using immunohistochemistry in stage II colon cancer patients.

This study aimed to evaluate tumor budding in a contemporary stage II colon cancer cohort from a screened population using IHC. We followed guidelines established by ITBCC and correlated H&E and IHC-based tumor budding using a four-tiered scoring system that included a Bd0 category. We investigated the potential of a cut-off to differentiate between high and low-grade tumor budding. The prognostic significance of tumor budding was examined by comparing time to recurrence and/or death between tumor budding groups while controlling for clinicopathological factors.

## Materials and methods

### Ethical statement

The reporting of this study follows the guidelines outlined in the Reporting Recommendations for Tumor MARKer prognostic studies (REMARK). The study adhered to the Declaration of Helsinki and received approval from The Regional Committees on Health Research Ethics for Southern Denmark (S-20190164), with dispensation from obtaining informed consent from the study patients. No patients were excluded based on registration in the Danish Registry of Tissue Utilization.

### Patients and tissue

This population-based study included 493 patients who underwent curative surgical resection of UICC stage II colon cancer between 2014 and 2016 in the Region of Southern Denmark. The patients were sourced from a screened population across four hospitals identified using the Danish Colorectal Cancer Group database and the Danish Pathology System. None of the patients included received neoadjuvant chemotherapy, and they had no history of colon cancer or any malignant disease (except non-melanoma skin cancer) within the 10 years leading up to the diagnosis of colon cancer. Patients who received postoperative adjuvant chemotherapy were included in the study, comprising 69 individuals, corresponding to 14% of the cohort. Further information on patient selection is described in detail elsewhere [28].

A retrospective histopathological characterization of the tumors was performed, coupled with a comprehensive review of medical records encompassing surgical details, follow-up information, and survival data. Details of the baseline clinicopathological characteristics have previously been presented [28].

All archived formalin-fixed, paraffin-embedded tissue blocks and slides originally utilized for routine diagnostic purposes were obtained from the four pathology departments in the Region of Southern Denmark. The number of tumor-containing tissue blocks per patient varied from 2 to 48, with a mean of 7. Histologic sections of 4-um thickness were cut from the tumor block with the highest degree of H&E-based tumor budding, and consecutive sections were stained with H&E and pan-cytokeratin, respectively.

### Immunohistochemistry

Immunohistochemical staining was performed automatically on a DAKO Autostainer Link 48 platform (DAKO, Glostrup, Denmark) as described elsewhere [23]. In short, the primary antibody used was mouse monoclonal anti-cytokeratin (clone AE1AE3, code M3515, DAKO, Glostrup, Denmark) diluted at 1:250. Following deparaffination and rehydration, antigen retrieval was performed using Envision Target Retrieval Solution (DAKO, Glostrup, Denmark) at pH 9 and 97 °C for 20 min. Slides were treated with EnVision FLEX Peroxidase-Blocking Reagent (DAKO) for 5 min to inhibit endogenous peroxidase, followed by a 30-min incubation with primary antibody at room temperature. Amplification was achieved using Envision Flex + Mouse (Linker) (DAKO, Glostrup, Denmark) for 20 min. Detection of bound antibodies was carried out using Envision FLEX/HRP (DAKO, Glostrup, Denmark) and visualized with Envision FLEX DAB (DAKO, Glostrup, Denmark) and Chromogen. Hematoxylin served as the counterstain.

### Evaluation of tumor budding

Tumor budding is defined as single tumor cells or clusters of up to four cells budding of the primary tumor [5]. The evaluation of tumor budding was done following the ITBCC guidelines, and all diagnostic H&E slides were reviewed at low power to identify the tumor block with the highest degree of budding at the invasive front [5]. Based on two consecutive sections from this tissue block, tumor budding was assessed using both H&E and IHC, following the same method: Ten individual fields were scanned at medium power, and tumor buds were counted in the hotspot area normalized to the field size of 0.785 mm^2^ using a Leica HC microscope. The tumors were categorized based on the proposed categories by ITBCC, including a separate category for Bd0 tumors. Consequently, a four-tiered scoring system, as suggested by Zlobec et al. [25], was implemented, classifying them into Bd0 (zero) 0 buds, Bd1 (low) 1–4 buds, Bd2 (intermediate) 5–9 buds, and Bd3 (high) ≥ 10 buds. We enumerated up to 100 buds and assigned a count of 100 to tumors exceeding this number. The pan-cytokeratin-stained tumor budding cells were required to show cytoplasmatic positivity and a clearly defined hematoxylin-stained nucleus to distinguish the cells from apoptotic bodies and cellular debris.

Caution was exercised when assessing tumor budding in regions exhibiting significant inflammation in order to differentiate true buds from mechanically fragmented glands and not erroneously count these so-called pseudo buds as genuine tumor buds.

### Intra- and interobserver agreement

The assessment of tumor budding was conducted by one observer, MPK, while SKF contributed to the interobserver evaluation. The observers scored the tumors independently of each other and were blinded to former bud count, as well as clinical and histopathological information. The intra- and interobserver reproducibility was assessed on 50 randomly selected tumor slides from both T3 and T4 tumors.

### Statistics

Summary statistics included mean and standard deviation (normal-distributed variables) or median and interquartile range (non-normal-distributed variables). Categorical variables are presented as numbers and percentages. Analyses of associations between tumor budding categories and clinicopathological characteristics used the chi-squared test or Fisher’s exact test, where appropriate. The Wilcoxon rank-sum test or Wilcoxon signed-rank test was employed for independent or matched continuous variables, respectively.

Weighted kappa statistics were used to determine the intra- and interobserver agreement between the tumor budding categories. A comparison of the tumor budding categorization assessed by H&E or IHC was performed using descriptive statistics and visualized using a scatter plot and a Bland–Altman plot.

For the prognostic evaluation, a receiver operating characteristic (ROC) curve analysis with either recurrence or death as an endpoint was performed to determine a clinically relevant cut-off score for IHC-evaluated tumor budding.

Time to recurrence (TTR) was defined as the time from surgery to the date of local or distant recurrence of colon cancer or the date of death from colon cancer. Recurrence-free survival (RFS) was defined as the time from surgery to the date of local or distant recurrence or death from any cause, whichever occurred first. Overall survival (OS) was defined as the time from surgery to death from any cause or end of follow-up. If no events occurred, all records were censored either at the point of loss to follow-up (*n* = 2) or at the end of the study period (May 15th, 2023). Events of metachronous cancer in the follow-up period were not considered a censoring event in the analyses [29].

Kaplan–Meier curves and log-rank tests were used to test for differences in survival times by the tumor budding groups. Uni- and multivariable Cox regression models were used to estimate hazard ratios (HR) and 95% confidence intervals (CIs). Bd0 was used as the reference group.

The multivariable analysis was adjusted for potential confounders identified by a previously published causal-directed acyclic graph (DAG) [28] and included the T category, mismatch repair (MMR) status, and histologic type. Multivariable analyses were conducted on complete cases (*n* = 492) due to minimal missing data (MMR status not assessed in one tumor).

Scaled Schoenfeld residuals checked the proportional hazard assumption for each regression analysis and did not violate it.

All analyses were carried out using Stata software (version 18.0 BE). All data were recorded in a Research Electronic Data Capture (REDCap®) database with an automatically generated entry check via the Open Patient Data Explorative Network (OPEN) organization. *P*-values of < 0.05 were considered to be statistically significant.

## Results

### Patient characteristics and clinicopathological data

The study included 497 patients with complete clinicopathological data and available diagnostic slides. Four cases failed to complete the IHC evaluation due to technical reasons. The analyses were conducted on 493 cases with complete tumor budding evaluation by both H&E and IHC.

Out of the 493 patients, 43 (9%) experienced a recurrence, and 175 patients died, of whom 27 died from colon cancer. The median follow-up time was 6.7 years (range 0.4–9.3 years).

### Tumor budding assessment by H&E and IHC

**Fig. 1:**
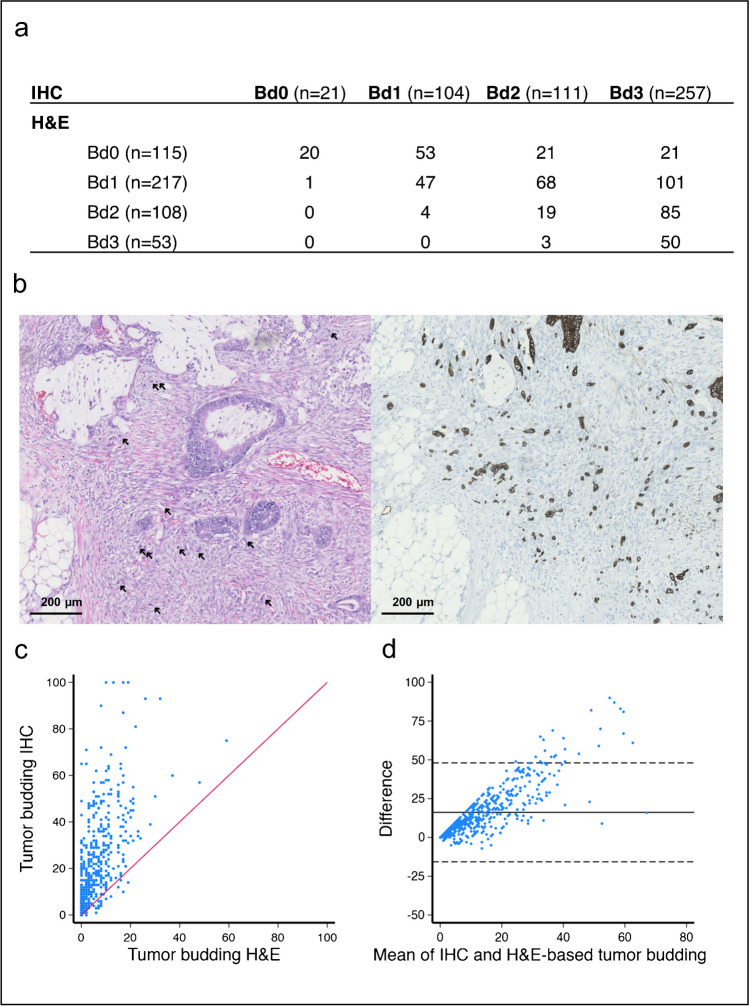
Comparison of H&E and IHC-based methods for evaluating tumor budding on consecutive tumor slides. (**a**) Consistency table showing the correlation between a four-tired tumor budding categorization system assessed by H&E and pan-cytokeratin IHC. (**b**) Comparative images of tumor budding at the invasive front in a stage II colon cancer. Sections were stained with standard H&E on the left (tumor buds are marked with arrows), and the same region is depicted on the corresponding slide stained with IHC on the right. IHC enables the identification of budding cells that were not visible with H&E staining. (**c**) Scatter plot showing the correlation between the tumor budding counts assessed using H&E versus IHC. (**d**) Bland–Altman plot displaying the difference (*y*-axis) and average (*x*-axis) of tumor budding counts based on H&E and IHC stained tumor slides. The solid horizontal line is the mean (16), and the dashed line represents the 95% limits of agreement (upper line = mean + 1.96 × SD, lower line = mean – 1.96 × SD)

The distribution of tumor budding in categories evaluated by H&E was as follows: 115 (23%) Bd0, 217 (44%) Bd1, 108 (22%) Bd2, and 53 (11%) Bd3, whereas assessment by IHC resulted in 21 (4%) Bd0, 104 (21%) Bd1, 111 (23%) Bd2, and 257 (52%) Bd3. Evaluation by IHC classified more tumors as Bd3 than by H&E (Fig. 1a). All tumors examined exhibited positive pan-cytokeratin immunohistochemistry. The tumor cells were prominently highlighted and readily discernible, thereby facilitating their assessment (Fig. 1b). The H&E-based evaluation resulted in a median of 4 buds (range 0–59) and involved a review of an average of 7.2 slides. As expected, the tumor bud count assessed by IHC was significantly higher (*p* < 0.01) and showed a median of 17 (range 0–100) (Fig. 1c). The IHC tumor bud count was, on average, 16 buds higher and the disparity between the staining methods escalated with increasing bud count, as illustrated in the Bland–Altman plot in Fig. 1d.

### Intra- and interobserver variability in the four-tiered grading system

Regarding tumor budding estimation, both intraobserver agreement using H&E staining (Kappa 0.76) and IHC-based evaluation (Kappa 0.76) demonstrated substantial agreement. Moreover, interobserver agreement showed improvement from H&E-based evaluation (Kappa 0.64) to IHC-based evaluation (Kappa 0.68), remaining substantial.

### Tumor budding cut-off determination

The ROC-derived thresholds were investigated and revealed no clear cut-off for recurrence discrimination in the distribution of tumor budding counts within this cohort (Supplementary Fig. 1). The area under the curve (AUC) was 0.52, indicating the method did not have significant discrimination capacity to differentiate between recurrence and non-recurrence, as it was not significantly higher than the value of 0.5 where the prediction ability would equal a random guess.

Given the outcomes yielded from the ROC curve analysis, we have endeavored to identify an optimal threshold for evaluating tumor budding through the application of IHC. This pursuit aims to establish a suitable cut-off point for accurate classification and prognostic assessment based on IHC-based tumor budding measurements. This determination involved considering multiple factors, such as the traditional Youden index and Liu’s index, along with selecting a threshold based on existing literature. Using the Youden index, the resulting cut-off value was 38, yielding a sensitivity of 16% and a specificity of 96%. Considered together with Liu´s index as well as the previously published cut-off value of 25 buds/0.785mm^2^ by Prall et al. [20], none had been identified as the optimal differentiating threshold (Supplementary Table 1).

The ROC-derived thresholds were also examined for their association with mortality as an endpoint, with the AUC yielding a similar result (0.52).

### Characteristics of the Bd0 tumors

Among the 493 patients included in the study, 21 patients were identified as having a complete absence of tumor budding and categorized as Bd0 based on IHC. On the corresponding H&E slide, the Bd0 comprised 115 tumors. Twenty tumors classified as Bd0 on IHC were included in the H&E-based Bd0 group (Fig. 1a).

The IHC-based Bd0 tumors differed significantly from tumors exhibiting budding concerning MMR status, histologic subtype, and postoperative adjuvant chemotherapy (*p* = 0.04, *p* = 0.01, and *p* = 0.05, respectively) (Table 1). Twenty tumors (95%) showed microsatellite stability (MSS). It appeared that IHC-based Bd0 tumors were frequently located in the left side of the colon (67%) and had a mucinous phenotype (38%), although not consistently. There was a higher prevalence of T4 tumors in the IHC-based Bd0 group compared to tumors with budding; however, this difference was not statistically significant. No lymphatic invasion was observed among the IHC-based Bd0 tumors. Despite a high degree (29%) of venous invasion among IHC-based Bd0 tumors, no recurrences were observed in the group. However, it is worth noting that six of the patients (29%) received postoperative adjuvant chemotherapy. This percentage is partly due to the high proportion of T4 tumors, as four out of five patients with T4 were treated.

**Table 1:** Patient characteristics and correlation of tumor budding status with clinicopathological data in UICC stage II colon cancer (*n* = 493). Tumor budding is evaluated by H&E and IHC. Data are n (%) unless otherwise stated

	**Tumor budding**							
	**IHC**				**H&E**			
	Bd0	Bd	Total	*p*-value	Bd0	Bd	Total	*p*-value
	*n* = 21	*n* = 472	*n* = 493		*n* = 115	*n* = 378	*n* = 493	
**Age at surgery**								
Mean (SD)	70 (9)	73 (10)	73 (10)	0.18	73 (10)	73 (10)	73 (10)	0.90
**Examined lymph nodes**								
Median [IQR]	28 [20 52]	26 [19 37]	26 [19 37]	0.39	30 [19 43]	25 [19 37]	26 [19 37]	0.04*
**Sex**								
Male	10 (48)	220 (47)	230 (47)	0.93	57 (50)	173 (46)	230 (47)	0.48
Female	11 (52)	252 (53)	263 (53)		58 (50)	205 (54)	263 (53)	
**Screening**								
Yes	6 (29)	93 (20)	99 (20)	0.32	23 (20)	76 (20)	99 (20)	0.98
No	15 (71)	379 (80)	394 (80)		92 (80)	302 (80)	394 (80)	
**Surgical approach**								
Acute	2 (10)	46 (10)	48 (10)	1.00	9 (8)	39 (10)	48 (10)	0.43
Elective	19 (90)	426 (90)	445 (90)		106 (92)	339 (90)	445 (90)	
**Anastomotic leakage**								
Yes	1 (5)	15 (3)	16 (3)	0.51	4 (3)	12 (3)	16 (3)	0.77
No	20 (95)	457 (97)	477 (97)		111 (97)	366 (97)	477 (97)	
**Tumor localization**								
Right	7 (33)	242 (51)	249 (51)	0.11	52 (45)	197 (52)	249 (51)	0.20
Left	14 (67)	230 (49)	244 (49)		63 (55)	181 (48)	244 (49)	
**Histological type**								
Glandular	13 (62)	371 (79)	384 (78)	0.01*	75 (65)	309 (82)	384 (78)	< 0.01*
Mucinous	8 (38)	62 (13)	70 (14)		29 (25)	41 (11)	70 (14)	
Low differentiated	0 (0)	39 (8)	39 (8)		11 (10)	28 (7)	39 (8)	
**Tumor differentiation**								
Well, moderate	21 (100)	433 (92)	454 (92)	0.40	104 (90)	350 (93)	454 (92)	0.45
Poor	0 (0)	39 (8)	39 (8)		11 (10)	28 (7)	39 (8)	
**T category**								
pT3	16 (76)	419 (89)	435 (88)	0.08	99 (86)	336 (89)	435 (88)	0.41
pT4	5 (24)	53 (11)	58 (12)		16 (14)	42 (11)	58 (12)	
**Venous invasion**								
Yes	6 (29)	109 (23)	115 (23)	0.56	24 (21)	91 (24)	115 (23)	0.48
No	15 (71)	363 (77)	378 (77)		91 (79)	287 (76)	378 (77)	
**Lymphatic invasion**								
Yes	0 (0)	25 (5)	25 (5)	0.62	6 (5)	19 (5)	25 (5)	0.94
No	21 (100)	447 (95)	468 (95)		109 (95)	359 (95)	468 (95)	
**Perineural invasion**								
Yes	1 (5)	57 (12)	58 (12)	0.49	3 (3)	55 (15)	58 (12)	< 0.01*
No	20 (95)	415 (88)	435 (88)		112 (97)	323 (85)	435 (88)	
**MMR**^**a**^								
pMMR	20 (95)	350 (74)	370 (75)	0.04*	84 (74)	286 (76)	370 (75)	0.67
dMMR	1 (5)	121 (26)	122 (25)		30 (26)	92 (24)	122 (25)	
**Postoperative adjuvant chemotherapy**								
Yes	6 (29)	63 (13)	69 (14)	0.05*	16 (14)	53 (14)	69 (14)	0.98
No	15 (71)	409 (87)	424 (86)		99 (86)	325 (86)	424 (86)	
**Postoperative recurrence**								
Yes	0 (0)	43 (9)	43 (9)	0.24	9 (8)	34 (9)	43 (9)	0.70
No	21 (100)	429 (91)	450(91)		106 (92)	344 (91)	450 (91)	

*MMR*, mismatch repair; *pMMR*, mismatch repair proficient; *dMMR*, mismatch repair deficient; *Bd0*, tumors without budding; *Bd*, tumors exhibiting budding

^a^Numbers may vary due to missing data for one patient

^*^Statistical significance (*p* < 0.05)

In comparison, the H&E-based Bd0 tumors demonstrated variances from tumors exhibiting budding solely in relation to the examination of lymph nodes, histological subtype, and perineural invasion.

Our analysis revealed that a higher number of lymph nodes were examined in the H&E-based Bd0 tumors, which presents an unexpected finding. The H&E-based Bd0 tumors were more frequently characterized by mucinous phenotype and demonstrated a lower incidence of perineural invasion. This aligns with previously documented correlations between tumor budding, histological subtype, and perineural invasion [28]. Apart from these discrepancies, the H&E-based Bd0 tumors closely resemble budding tumors, suggesting a less prominent distinction compared to the IHC-based approach. The other characteristics observed in the IHC-based Bd0 tumors were not observed in the H&E-based Bd0 tumors.

The application of IHC revealed tumor buds that may not have been visible. Therefore, the IHC-based Bd0 group can be considered as true Bd0 tumors.

### IHC-based Bd0 adds prognostic value

**Fig. 2:**
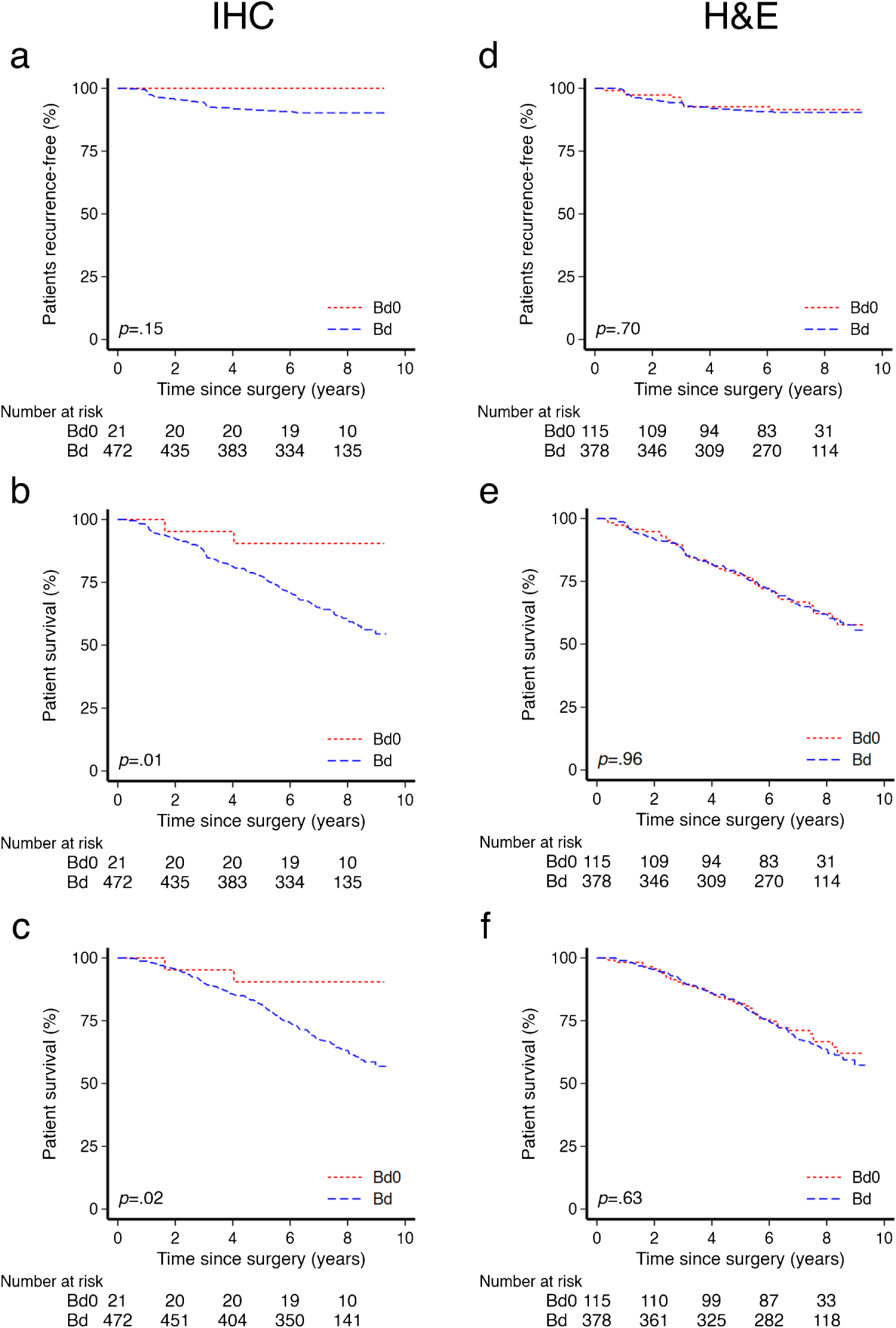
Kaplan-Meyer survival curves illustrating the association between tumors with (Bd) and without (Bd0) budding and survival endpoints in a stage II colon cancer cohort (*n* = 493) for IHC (**a**-**c**) and H&E-based evaluation (**d**-**f**). Time to recurrence (TTR) (**a**,**d**), recurrence-free survival (RFS) (**b**,**e**), and overall survival (OS) (**c**,**f**)

Survival analyses were performed on the cohort, grouping the patients based on whether there was tumor budding or not. The 5-year rate of RFS was 90% in the IHC-based Bd0 group compared to 78% in the tumors with budding, while the corresponding 5-year rate of OS was 90% and 82%, respectively (Fig. 2). No recurrences occurred in the IHC-based Bd0 group in contrast to 43 (9%) in the tumor budding group. The two groups exhibited statistically significant differences in survival functions for RFS (*p* = 0.01) and OS (*p* = 0.02), while the difference did not reach statistical significance for TTR (*p* = 0.15). The results from the uni- and multivariable Cox regression analyses are presented in Table 2. The presence of tumor budding was significantly associated with reduced RFS (HR = 4.95, 95% CI 1.23–19.96, *p* = 0.02) and OS (HR = 4.51, 95% CI 1.12–18.18, *p* = 0.03) compared to no tumor budding. The presence of tumor budding maintained a significant and adverse effect on survival outcomes RFS (HR = 5.19, 95% CI 1.27–21.16, *p* = 0.02) and OS (HR = 4.47, 95% CI 1.10–18.27, *p* = 0.04) when correcting for MMR status, T category, and histologic type.

**Table 2:** Cox regression analysis for recurrence-free survival and overall survival (*n* = 493). Multivariable analysis adjusted for mismatch repair status, T category, and histologic type (*n* = 492)

**IHC Univariable analysis**							
		**Recurrence-free survival **		**Overall survival **			
**Tumor budding**	*n (%)*	HR (95% CI)	*p*-value	HR (95% CI)	*p*-value		
No (Bd0)	21 (4)	ref		ref			
Yes (Bd)	472 (96)	4.95 (1.23-19.96)	0.02*	4.51 (1.12-18.18)	0.03*		
**IHC Multivariable analysis**							
		**Recurrence-free survival**		**Overall survival**			
**Tumor budding**	*n (%)*	HR (95% CI)	*p*-value	HR (95% CI)	*p*-value		
No (Bd0)	21 (4)	ref		ref			
Yes (Bd)	471 (96)	5.19 (1.27-21.16)	0.02*	4.47 (1.10-18.27)	0.04*		
**H&E Univariable analysis**							
		**Time to recurrence**		**Recurrence-free survival**		**Overall survival**	
**Tumor budding**	*n (%)*	HR (95% CI)	*p*-value	HR (95% CI)	*p*-value	HR (95% CI)	*p*-value
No (Bd0)	115 (23)	ref		ref		ref	
Yes (Bd)	378 (77)	1.15 (0.55-2.40)	0.70	1.01 (0.72-1.42)	0.96	1.09 (0.76-1.56)	0.63
**H&E** **Multivariable analysis**							
		**Time to recurrence**		**Recurrence-free survival**		**Overall survival**	
**Tumor budding**	*n (%)*	HR (95% CI)	*p*-value	HR (95% CI)	*p*-value	HR (95% CI)	*p*-value
No (Bd0)	115 (23)	Ref		ref		ref	
Yes (Bd)	378 (77)	1.27 (0.59-2.74)	0.53	1.00 (0.71-1.41)	1.00	1.08 (0.75-1.55)	0.69

A subgroup analysis of the H&E-based categorization did not show any significant differences between patients with budding tumors and those without in terms of survival endpoints (Fig. 2). No differences were observed in the uni- or multivariable Cox regression analysis, with almost identical hazard rates being achieved (Table 2).

## Discussion

In this retrospective, population-based cohort study, we highlighted tumor budding in a contemporary stage II colon cancer cohort using IHC. The identification of tumor buds increased dramatically using IHC, with tumors categorized as Bd3 showing a five-fold increase. The average bud count was 16 cells higher with IHC, and the differences between the two approaches escalated as the bud count increased. In 21 tumors, a complete absence of tumor budding based on IHC was observed. Remarkably, during the follow-up period, none of these patients experienced recurrences and demonstrated a significantly increased RFS as well as OS.

Finding a clinically applicable cut-off point for tumor budding in this study proved to be a significant challenge. Other studies have used different cut-off values to categorize IHC-based tumor budding. Prall et al. [20] found a cut-off of 25 tumor buds and reported a strong association between high-grade tumor budding and poor prognosis in stage I/II colorectal cancer examining a field of view measuring 0.785 mm^2^, as suggested by the ITBCC guidelines while incorporating up to five cells within their definition of tumor budding. Karamitopoulou et al. [24] determined a cut-off of 10 tumor buds for prognostic subgroups across 10 HPFs in colorectal cancer. Quantitative scoring methods with no cut-offs have also been used, with Horcic et al. [21] showing an exponential effect on the risk of death with increasing numbers of tumor buds in stage II colon cancer. Rieger et al. [22] found significant associations between continuous peritumoral tumor budding scores both in a hotspot and in 10 HPF and disease-free survival in all stages of colorectal cancer, but the association was lost when evaluated by pre-defined cut-off scores. The different approaches and cut-off values proposed in similar studies reflect that finding a cut-off point for IHC-based tumor budding is not a straightforward task. Our results contribute to this discussion. In such circumstances, translating a prognostic biomarker into clinical practice becomes challenging, as clear guidelines for a biomarker must be in place before it can be clinically applied.

Our recent study demonstrated the prognostic significance of high-grade tumor budding, as assessed by H&E staining, in the same patient cohort. We used the ITBCC guidelines and their recommended three-tiered classification system. With the use of H&E staining, we were able to distinguish different prognostic outcomes based on the established cut-off points. However, it seemed that the determination of tumor budding using IHC did not show the same pattern. There is no established criteria for cut-off values, and the literature explores different approaches and thresholds. In this study, we attempted to determine a cut-off value, but we were unable to find one that seemed clinically relevant. Consequently, this discrepancy led to the exclusion of a comparable classification between H&E and IHC staining, and it required a broader analysis of IHC tumor budding, where the distinction between budding and non-budding was made. This revealed intriguing prognostic significance associated with IHC-based Bd0.

Our findings indicate that the Bd0 subgroup is associated with a complete absence of recurrences, suggesting that Bd0 carries a 100% predictive value for the absence of recurrences. The unique feature of the Bd0 group is its composition of patients who do not align with the low-risk category based on established risk factors. When examining Table 1, a distinct morphological profile of the IHC-based Bd0 tumors is not readily apparent, although these tumors were more likely to be of a mucinous type and show pMMR status. In future studies, it would be prudent to investigate the presence of inflammation in these tumors, particularly along with their molecular characteristics.

The level of agreement in categorizing tumor budding among different observers varies across studies [15, 21, 24]. Kai et al. [15] showed that more experienced pathologists tend to assign higher tumor budding grades. In our results, the less experienced observer had a higher bud count, regardless of the staining approach (data not shown). Despite this, the interobserver agreement was deemed acceptable regardless of the staining method. The implementation of IHC demonstrated a slight improvement in the interobserver agreement, although a significant advantage for IHC over H&E was not observed, which aligns with findings from other studies [30, 31]. Therefore, it is essential to recognize that there may still be variability among observers in the assessment of IHC, and this variability remains significant [31].

The size of the field of view is an important consideration when evaluating tumor budding. The ITBCC recommendations include the possibility of normalizing the field of view to a standard area of 0.785 mm^2^. However, this normalization may result in an underestimation of the bud count if the budding cells are not evenly distributed across the field of view [32]. This effect is expected to be more pronounced when using IHC, which typically yields higher bud counts. Furthermore, this presents challenges when comparing findings with other studies, as previous studies using the 1HPF method often employ smaller fields of view, such as 0.238 mm^2^ [22] and 0.49 mm^2^ [21]. Therefore, caution must be exercised when extrapolating bud counts from other studies.

Our results demonstrate that the use of IHC in comparison to H&E-stained sections detects three to four times more buds. These findings must be interpreted with caution, not just assuming that the IHC-based approach simply just facilitates the visualization of budding cells. Utilizing immunohistochemistry (IHC) for assessing tumor budding presents challenges in interpreting morphology and avoiding potential pitfalls, including pseudobudding. Distinguishing true buds from mechanically fragmented glands is difficult. True tumor buds infiltrate the peritumoral stroma, while pseudobuds are surrounded by inflammatory cells and typically found near fragmented glands caused by reactive processes like inflammation and glandular disruption [18, 33]. The presence of pseudobudding can lead to misleading results when using IHC staining, as individual cytokeratin-positive cells may be mistakenly counted as true tumor buds, artificially inflating the bud count. Caution is advised when evaluating tumor budding in areas with significant inflammation. The use of H&E staining is essential in such cases and cannot be substituted by IHC. H&E and IHC must complement each other, and perhaps we should not place excessive emphasis on the transferability of the H&E method to IHC but rather explore the alternative possibilities inherent in IHC. In the current era where artificial intelligence has gained significant attention, there have been numerous efforts to develop semi-automated methods for assessing morphological characteristics, such as tumor budding [34, 35]. In this regard, utilizing IHC may serve as a viable substitute for H&E staining in constructing these applications. However, relying solely on IHC for these assessments may pose some challenges as moderate agreement between observers has been reported, with complete agreement observed for only 34% of 3000 tumor bud candidates in a recent study [31]. Therefore, it is important to emphasize the synergy between IHC and H&E, as it offers a more comprehensive perspective.

In conclusion, prognostic markers need to exhibit appropriate levels of sensitivity and specificity to ensure clinical relevance. In this retrospective study, using a contemporary stage II colon cancer cohort, we were not able to find such a meaningful cut-off based on IHC-evaluated tumor budding.

The successful adoption of the Bd0 category using IHC is of prognostic significance and mandates the need for further independent studies to gather an adequate amount of data. Due to the limited number of tumors (*n* = 21) not exhibiting budding, our ability to draw significant conclusions is constrained. Nevertheless, our research findings indicate that Bd0 tumors display a lower level of aggressiveness in colon cancer compared to tumors that exhibit any degree of budding, and this is significant in a clinical setting when making the decision regarding adjuvant chemotherapy.

## Supplementary Information

Below is the link to the electronic supplementary material.

Supplementary file1 (DOCX 1270 KB)

Supplementary file2 (PDF 182 KB)

## Data Availability

The datasets generated and analyzed during the current study are available from the corresponding author upon reasonable request.
