# Supplementary material for: Immunohistochemical analysis of tumor budding in stage II colon cancer: exploring zero budding as a prognostic marker
Source: Virchows Arch. 2024 Jul 8;485(4):691–701. doi: 10.1007/s00428-024-03860-2 (PMC11522105; doi:10.1007/s00428-024-03860-2)
Supplement: Supplementary file 1 — Supplementary file1 (DOCX 1270 KB) [file 428_2024_3860_MOESM1_ESM.docx]

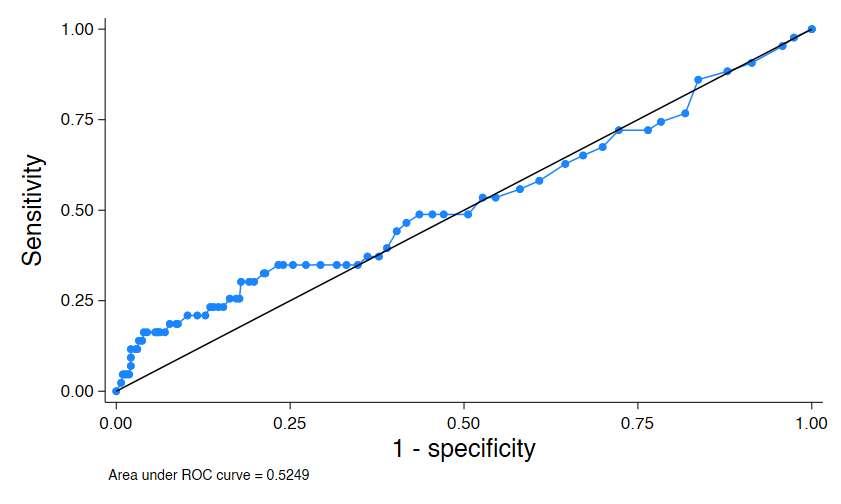


**Supplementary Fig. 1**

Receiver Operating Characteristic (ROC) curve used to determine the optimal cut-off value for distinguishing between Bd low and Bd high tumors with recurrence as an end-point
