# Supplementary material for: Immunohistochemical analysis of tumor budding in stage II colon cancer: exploring zero budding as a prognostic marker
Source: Virchows Arch. 2024 Jul 8;485(4):691–701. doi: 10.1007/s00428-024-03860-2 (PMC11522105; doi:10.1007/s00428-024-03860-2)
Supplement: Supplementary file 2 — Supplementary file2 (PDF 182 KB) [file 428_2024_3860_MOESM2_ESM.pdf]

Supplementary Table 1. Sensitivity, specificity, positive predictive value (PPV), and negative predictive value (NPV) of recurrence in a stage II colon cancer population (n=472, excluding Bd0 patients) for each IHC evaluated tumor budding cut-off point.

**Cut-off**	True positive (no.)	True negative (no.)	False positive (no.)	False negative (no.)	Sensitivity (%)	Specificity (%)	PPV (%)	NPV (%)
14 (Lui)	21	242	187	22	49	56	10	92
25 (Prall et al.)	13	352	77	30	30	82	14	92
38 (Youden)	7	412	17	36	16	96	29	92
